# Screening for New Cosmeceuticals from Brown Algae *Fucus vesiculosus* with Antioxidant and Photo-Protecting Properties

**DOI:** 10.3390/md20110687

**Published:** 2022-10-31

**Authors:** Ditte B. Hermund, Hanna Torsteinsen, Julia Vega, Félix L. Figueroa, Charlotte Jacobsen

**Affiliations:** 1 National Food Institute, Technical University of Denmark, Kemitorvet 202, DK-2800 Kgs. Lyngby, Denmark; 2 Instituto Universitario de Biotecnología y Desarrollo Azul (IBYDA), Departamento de Ecología, Universidad de Málaga, Campus Universitario de Teatinos s/n, 29071 Málaga, Spain

**Keywords:** antioxidant activity, effective solar absorption radiation, ethanolic extract, water extract, extract photoprotection index, macroalgae, Mansur method, total phenolic content, skin care, UV–vis absorption spectra

## Abstract

Phlorotannins play a role in biological functions to protect the cells against UV and oxidative damage in brown algae. We hypothesized that these compounds can function as photo-protectors and antioxidants in skin care formulations. Two types of extracts (water (FV-WE) and 67% *v*/*v* ethanol (FV-EE)) from *Fucus vesiculosus* were obtained with a phlorotannin content between 7−14% in dry extract. Exposure to sun light during growth was included as a factor on the phlorotannin content but did not influence the phlorotannin content. However, green colored *F. vesiculosus* had lower total phenolic content (TPC) (FV-WE = 6.9 g GAE 100 g^−1^ dw, FV-EE = 7.8 g GAE 100 g^−1^ dw) compared to those with a yellow/brownish color (FV-WE = 10.4–13.7 g GAE 100 g^−1^ dw, FV-EE = 11.2–14.0 g GAE 100 g^−1^ dw). UVA and UVB photo protective capabilities of the extracts through different biological effective protection factors (BEPFs) were evaluated using in vitro methods; the Mansur method for sun protection factor (SPF) and calculation of effective solar absorption radiation (%ESAR) to determine SPF and UVA protection factor (UVA-PF) of the extract and in seaweed enriched lotion. The SPF was negligible, when evaluating FV-WE in lotion (10 and 20% *w*/*w*). Moreover, %ESAR of the FV-WE showed SPF and some UVA-PF, but not enough to give sufficient SPF in lotions (10% *w*/*w*). It was concluded that the concentration of UV protecting compounds in the extracts was too low to and that further fractionation and purification of phlorotannins is needed to increase the SPF.

## 1. Introduction

Sun light is the main source of ultraviolet (UV) radiation, which consists of UVA (320–400 nm), UVB (280–320 nm), and UVC (200–280 nm) rays. UVA rays have the longest wavelengths, followed by UVB, and UVC rays, which have the shortest wavelengths. While UVA and UVB rays are transmitted through the atmosphere, all UVC and some UVB rays are absorbed by the ozone layer. Hence, most of the UV rays reaching Earth are UVA with a small amount of UVB. Because of the shorter wavelength of UVB rays, these reaches the outer layer of your skin (the epidermis). UVA rays have a longer wavelength that can penetrate the middle layer of your skin (the dermis) [1]. Excessive exposure to UVA and UVB rays causes sunburn (erythema), persistent pigment darkening (PPD) and elevated alterations in skin connective tissue (collagen network) caused by oxidative stress in the skin due to high production of reactive oxygen species (ROS), which leads to premature skin aging (wrinkling). Moreover, UV is epidemiologically and molecularly linked to the three most common types of skin cancer [1]. Action spectrum is a tool used to describe the relative effectiveness of UVA and UVB rays in the induction of health usually expressed on a log scale.

To minimize the damaging effects of UV rays, antioxidant and photo-protective products can be used to protect the skin and minimize premature aging of the skin [2]. Sunscreen is an important part of a complete photo-protection strategy to ensure healthy skin and humans. Moreover, antioxidant therapy is a well-known way to overcome oxidative stress conditions in the skin. Currently, the erythema action spectrum is used for the in vitro SPF determinations, and PPD action spectrum for the UVA protection factor (UVA-PF). The European Union recommends both a critical wavelength of more than 370 nm and a UVA–PF of at least one third of the labelled SPF as the criterion for labelling as either UVA or broad-spectrum protection of UV-filters (ISO 24443:2012) [3].

Recently, there has been a major shift towards the use of natural ingredients, clean labeling and non-synthetic chemicals, mainly due to increased awareness of toxicity and chemical cocktails in cosmetic products [4]. Furthermore, sunscreens can be harmful to the environment, as organic sun filters damage coral reefs, and have been banned in specific areas of the world, e.g., oxybenzone and octinoxate [5,6]. This increased costumer awareness makes the search for new natural ingredients in sunscreen unavoidable.

A promising in vitro antioxidant potential of the brown seaweed *Fucus vesiculosus* has been established by several authors, as this seaweed contains high levels of bioactive compounds with high antioxidant activity [7,8,9]. As subtidal brown seaweed species, such as *F. vesiculosus*, are exposed to high levels of solar radiation, synthesis and accumulation of UV-absorbing components is a common biological response in this group of organisms to protect it against UV induced cell damage and oxidative stress [10]. Hence, this has spiked the interest of several researchers to investigate the potential of utilizing different seaweed components as a supplement to UVB and UVA-absorbing filters in sunscreen [10,11]. 

Phlorotannins constitute the major group of phenolic compounds in brown algae. One of the biological functions of phlorotannins is to protect the seaweed against UV damage by both UV-screen and antioxidant capacity. Phlorotannins are oligomers of phloroglucinol (1,3,5-trihydroxybenzene) units (1 PGU = 126 Da). The size ranges from 0.126–650 kDa but is most commonly between 10−100 kDa. The complexity and structural diversity increase with increasing numbers of PGUs [12,13]. The concentration of phlorotannins can range from 5–30% of dry weight [14]. This high concentration indicates their multifunctional role and importance in the seaweed, and the great potential for phlorotannin-rich extracts of *F. vesiculosus* as an antioxidant-rich UV protective ingredient in sunscreen [15,16,17].

Phlorotannins are polar compounds, which can easily be extracted using water or aqueous solutions of ethanol. Previous studies have successfully used water and 80% ethanol to obtain phlorotannin rich extracts from *F. vesiculosus* with phlorotannin content of 13% and 16% of dry weight, respectively [9]. However, the phlorotannin content is highly dependent on season and studies show highest content in late summer/early autumn. Moreover, extrinsic factors such as age of the seaweed, salinity, growth depth, etc., also play a role on the phlorotannin content [18]. 

As an alternative to the standardized in vitro SPF-testing [3], the European Cosmetic and Perfumery Association (COLIPA) developed and published an in vivo SPF test method, which became the blueprint of current use (ISO 24444:2019) [19,20]. However, in vivo testing is time consuming and very expensive. Hence, much effort has been devoted to develop in vitro techniques for assessing photo-protection against other acute damages and most of the chronic ones mediated by other wavelengths in the UV range, with known action spectra, which could be used for complementary evaluations of sunscreen testing and labelling of broad-spectrum photoprotective capability in vitro. This will also help differentiating the effectiveness of sunscreen formulations with similar SPF in the future.

Mansur et al. [21] developed a very simple mathematical equation which substitutes the in vitro method proposed by Sayre et al. [22,23,24], utilizing UV spectrophotometry and the following equation (Equation (1)):

(1) $$\mathrm{SPF}=\mathrm{CF}\overset{320}{\underset{290}{\sum }}\mathrm{EE}\left(\lambda \right)\times \;l\left(\lambda \right)\times \mathrm{Abs}\left(\lambda \right)$$

CF is the correction factor (10), Abs (λ) is the spectrophotometric absorbance measured from 290 to 320 nm. The value of EE(λ) × I(λ) is a constant, describing the relationship between the erythemal efficiency spectrum (EE) and the solar simulator intensity spectrum (I) at wavelengths from 290 to 320 nm [22].

Another way to evaluate photo protecting properties is to determine effective solar absorption radiation (ESAR, in %) which indicates the potential of, e.g., seaweed extracts, to absorb different UV wavelengths that can induce correspondingly different biological responses and the extract photoprotection index (EPI) in skin care emulsions (lotion), which is an analogue to SPF and UVA-PF [25].

The aim of the study was to evaluate the potential of phlorotannin-rich extracts as natural antioxidant and UV protecting compounds in skin care application. Hence, two types of extracts were obtained from brown algae *Fucus vesiculosus*; a water (FV-WE) and an ethanolic extract (67%, FV-EE). Different grow depths (G1 = 15–30 cm, G2 = 20–30 cm, G3 = 30–50 cm, G4 = 50–70 cm) were investigated to evaluate the influence of environmental factors on the total phlorotannin content and in vitro antioxidant activity of the extracts. For evaluation of photoprotecting properties, SPFs and other Biological Effective Protection Factors (BEPFs) were determined using in vitro methods. Comparison of claimed SPF vs. SPF obtained using the Mansur method, was performed on three commercial sunscreens (SPF15, 25 and 45). Then the Mansur method was used as a screening tool to determine the in vitro SPF of lotion with seaweed extract added. %ESAR was calculated to determine UV protecting properties of the extracts against UV-induced biological responses other than UVB. Lastly, %ESAR and EPI was calculated for extracts and in lotions with extract added. 

## 2. Results and Discussion

### 2.1. Sampling of F. vesiculosus

To evaluate how growth depth (and indirectly solar exposure) influences antioxidant activity and UV protective capacity, four different growth depths were chosen (Table 1).

Previous studies showed that the phenolic content of brown algae harvested from cold water areas like Denmark, is highest in late summer/early autumn [9]. The estimated phlorotannin content of the *F. vesiculosus* previously harvested at this location in late August was 5.9% per dry weight (data not shown). The quality assessment was performed subjectively by the company providing the seaweed samples and refers to the eating quality (degree of biofouling and epiphytes). G2, G3 and G4 were of the same high quality. The low-quality G1 was included in this study to evaluate other potential application platforms for seaweeds not suitable for human consumption. G1 and G2 had the similar growth depth (G1 = 15–30 cm and G2 = 20–30 cm), but different qualities (G1 = low and G2 = high). The color of the seaweed was also different as G3 had a more greenish color compared to the other groups.

### 2.2. Growth Depth Dependent Antioxidant and Phlorotannin Content of F. vesiculosus Extracts

Phlorotannins are the major phenolic compound in brown algae. Hence, the total phenolic content (TPC) was estimated using Folin Ciocalteu assay and gallic acid equivalent (GAE) for quantification. Moreover, in vitro antioxidant properties were determined for FV-WE and FV-EE in all four groups. Results are shown in Table 2. The results were used to evaluate the influence of growth depth and extraction solvent on the TPC and antioxidant potential of the extracts obtained in this study.

TPC ranged from 6.9 to 13.7 g GAE 100 g^−1^ dw for FV-WE, and 7.8 to 14.0 g GAE 100 g^−1^ dw for FV-EE. For both FV-EE and FV-WE, the highest TPC was found when *F. vesiculosus* was of high quality, had a yellow/brownish color, and was harvested from 20–30 cm water (G2), whilst *F. vesiculosus* of high quality, greenish color, and a growth depth of 30–50 cm (G3), had significantly (*p* < 0.05) lower TPC than all other groups. Low quality *F. vesiculosus* from 15–30 cm water (yellow/brownish color) (G1) and high-quality *F. vesiculosus* from 50–70 cm water (G4) (yellow/brownish color) had similar TPC of approximately 11 g GAE 100 g^−1^ dry matter (ranged from 10.4−11.9 g GAE 100 g^−1^ dry matter). 

The TPC found in the present study is in agreement with previous findings. Hermund et al. [9] reported 13.4 and 16.5 g GAE 100 g^−1^ dw for water and 80% ethanolic extracts from Danish *F. vesiculosus*, respectively. Honold et al. [8] investigated the TPC of water extracts of old and young segments of Icelandic *F. vesiculosus* and found 12.6 and 6.9 g GAE 100 g^−1^ dw, respectively. A stability trail was conducted to determine the degradation of phenolic compounds during storage or when stressed (heated to 100 °C for 5, 15 or 30 min). The results are included in supplementary materials (Figures S3 and S4). The phenolic compounds in FV-WE were heat stable up to the maximum time for heat exposure (30 min). Moreover, FV-WE and FV-EE (group G1) were stored at −20 °C for 12 months to evaluate the storage stability. The extracts were compared with extracts stored at −80 °C under the assumption that phenolics are stable at this temperature. Results showed no significant decrease in phenolic compounds between extracts stored at −20 °C and the control extracts stored at −80 °C. This indicates good stability of phlorotannins in the extracts. 

There was no significant (*p* > 0.05) difference between the TPC when using water or ethanol. This is dissimilar to other studies [7,8,9,16], who found significantly higher levels of TPC in ethanolic extracts compared to water extracts. Phenolic compounds are increasingly soluble in less polar solvents, and ethanol and acetone are hence the recommended solvents for extraction of polyphenolic compounds [7,8,16,26]. However, mannitol will be co-extracted using organic solvents like ethanol. Reducing sugars, like mannitol, are included in the determination of TPC using Folin Ciocalteu assay measuring all reducing compounds. Hence, an overestimation of TPC in FV-EE is plausible since no precipitation of mannitol was included in the purification of the extracts. Mannitol can also influence the antioxidant properties such as 1,1-diphenyl-2-picrylhydrazyl (DPPH) free radical scavenging capacity.

The DPPH free radical scavenging capacity ranged from an EC50 of 31.1 to 76.3 μg dw mL^−1^ in FV-WE and from 15.5 to 30.9 μg dw mL^−1^ in FV-EE. The ferrous iron chelating (FIC) ability ranged from an EC50 of 311.2 to 365.1 μg dw mL^−1^ in FV-WE, and from 625.3 to 1052.4 μg dw mL^−1^ in FV-EE. The term EC50 describes the concentration of a substance that provides half of the maximal response (50%) of a biological or chemical pathway, i.e., a low EC50 signifies higher antioxidant efficacy in the specific assay. FV-WE exhibited significantly lower DPPH radical scavenging capacity and significantly higher FIC ability compared to FV-EE. Between groups, only G3 was different from the other groups, except for the FIC ability of FV-EE, where no significant difference was found between the groups.

Farvin & Jacobsen [7] reported EC50 values of DPPH radical scavenging capacity of 8.3 and 9.9 μg dw mL^−1^ for water and ethanolic extracts of *F. vesiculosus*, respectively. This indicates higher radical scavenging activity compared to the results found in the present study. The FIC data reported in the study by Farvin & Jacobsen [7] was similar to the findings in the present study, with EC50 values of 128.6 and 1000.0 μg dw mL^−1^ for water and ethanolic extract, respectively.

**Figure 1:**
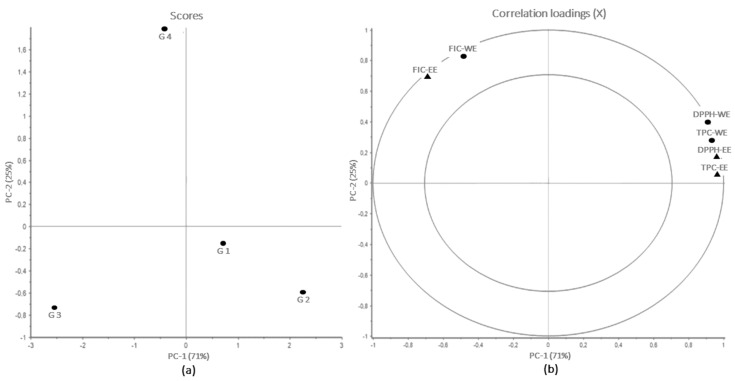
PCA scores (**a**) and loadings (**b**) for all four groups (G1–G4) for each extract type. Loading plot contains results from TPC and antioxidant properties; FV-WE (circles) and FV-EE (triangles).

A strong correlation between TPC and DPPH radical scavenging capacity, as shown in the principal component analysis (PCA) (Figure 1). The first two principal components (PC-1 and PC-2) explained 71% and 25% of the total variance in the data set, respectively. Due to the reverse meaning of EC50 values (i.e., higher values = lower antioxidant potency), the EC50 values are converted to negative values, to simplify interpretation of the correlations plot. Through the analysis of PC-1, it is possible to observe a strong correlation between TPC and DPPH radical scavenging capacity, both located far right on the PC-1 axis. This corresponds with the location of G3 on the scores plot (Figure 1a), as this group exhibited lower TPC and lower potency in scavenging DPPH radicals. Similarly, G1 and G2 exhibited lower FIC ability, and higher TPC and DPPH radical scavenging capacity, which also can be observed by comparing the scores and correlations loading plot. The same goes for G4, which exhibited the highest potency in FIC. These findings are similar to previous studies [8,14], which also found similar correlations between TPC and DPPH radical scavenging capacity in seaweed extracts. Moreover, the findings also clearly indicate that phenolic compounds are not the major contributors to FIC in seaweed extracts.

For FIC, this was the only case where the FV-WE exhibited higher efficiency compared to FV-EE, for all assays performed. This corresponds to the findings by several authors [7,8,26], found a higher FIC of water extracts compared to ethanolic or acetone extracts, indicating that water will to a higher extent co-extract components with high FIC, e.g., alginate [7].

The results display no clear pattern in TPC and antioxidant activities along the depth gradient. For TPC and DPPH radical scavenging capacity, only G3 performed significantly different (*p* < 0.05) compared to the other groups for both FV-WE and FV-EE. The FIC was the poorest in G2 for both FV-WE and FV-EE. These results therefore suggest that growth depth/solar exposure alone cannot explain the variation in TPC and in vitro antioxidant activity.

The lack of significant differences in TPC and antioxidant activity between the four groups could possibly be explained by the low variability in growth depths. Lann et al. [27] evaluated the correlation between depth and TPC, and found that TPC content decreased with depth for *Sargassum* sp. and *Turbinaria* sp. However, the depth zones in this study varied much more than in the present study, with growth depths of samples from 0–1 m; 1–3 m; and 3–6 m, whilst the growth depths in the present study ranged from 15–70 cm. 

The groups were also divided into low and high quality, related to food quality and depending on degree of biofouling. G1 was denoted as a low-quality seaweed (high level of biofouling and low food quality) and was included in this study to investigate whether the low-quality seaweed could be utilized for cosmeceutical purposes. Results showed no significant difference in TPC and antioxidant properties between low- and high-quality seaweed, except for G3. This indicates that low-quality seaweed unsuitable for human consumption could still be a valuable source of bioactive components. G3 was the only group that presented significantly lower TPC and radical scavenging capacity for both FV-WE and FV-EE. The color of G3 was green where the other groups had a brown/yellow color. Whereas the younger part of *F. vesiculosus*, the growing part, is greener, the older part is more brown [28]. Hemmi et al. [28] stated that higher nutrient content in the young part causes lower phenolic content. Hence, it is possible that G3 is a younger *F. vesiculosus*. This also corresponds well with the lower TPC of G3 compared to the other seaweed samples. Hence, other factors than growth depth seem to influence the biochemical composition of seaweed. Therefore, to fully understand the biochemical and biological variability, all the biotic and abiotic factors must be taken into account. 

At the end of the antioxidant and chemical characterization of the extracts, one high quality (G4) and one low quality (G1), yellow colored seaweed sample was chosen for further analysis.

### 2.3. Screening for Photo-Protecting Properties of Seaweed Extracts 

#### 2.3.1. Absorption Spectra of FV-WE and FV-EE

Absorption spectra of FV-WE and FV-EE from seaweed groups 1 and 4 were obtained from 280–400 nm and 400–700 nm (Figures S1 and S2). In general, FV-WE displays higher absorbance compared to FV-EE. This could be influenced by the higher level of dry matter in the FV-WE (FV-WE dm ≈ 1 %, FV-EE dm ≈ 0.7%). It appears that FV-WE also absorb in the UVA-range to a higher extent than FV-EE, suggesting that UVA-absorbing compounds are being co-extracted with water in FV-WE.

No prominent peaks were identified for either FV-WE or FV-EE in the UV-region, only a small indication of a peak around 330 nm. Schneider et al. [25] reported similar findings, i.e., no peak formation in the UV-region for brown seaweeds, whilst prominent peaks were visible in the absorption spectra for green and especially red seaweed. They found that red algae *P. umbilicalis* displayed the most prominent peak (320–340 nm), which was found to be caused by mycosporine-like amino acids (MAAs), which are normally associated with red algae.

Absorption spectra in the visible light range (400–700 nm), showed that both FV-WE and FV-EE absorb light in the visible light range. 

There is a small peak formation at around 650 nm, which is slightly more prominent in FV-EE. Moreover, FV-WE has indication of a peak around 450 nm. Schneider et al. [25] also reported peak formation between 600–700 nm for both green, red and brown seaweeds, coinciding with the absorption peaks of photosynthetic and protective pigments such as chlorophyll a, b, and c, phycobiliproteins and carotenoids. Schneider et al. [25] reported peak formation at around 450 nm, which indicates the presence of fucoxanthin. A previous study by Poyato et al. [29] found higher content of carotenoids incl. fucoxanthin in water extract (WE, 3.9 ± 0.9 mg mg^−1^ dry extract) compared to acetonic extract (AE, 0.8 ± 0.1 mg mg^−1^ dry extract) from *F. vesiculosus*. Hence, the findings could most likely be verified by determining the different pigments in the extracts used in the present study, e.g., fucoxanthin and chlorophylls.

#### 2.3.2. SPF of Commercial Sunscreens for Testing the Mansur Method

The Mansur method has spiked some controversy due to the wrong interpretation of results, methodology and low accuracy [11,21]. In the present study, the Mansur method was tested using three commercial sunscreens (Table 3) with claimed SPF of 15, 25 and 45 (sunscreen A, B and C, respectively), to ensure correct interpretation of the results when applying the method on unknown samples. The commercial products were measured spectrophotometrically and calculated by applying the Mansur equation (Equation (1), Table 4).

The measured SPF for sunscreen A was close to the claimed SPF (16 vs. 15). However, for sunscreen B and C, the measured SPF was well below the claimed SPF, with SPF 16 and 21 measured for sunscreen B and C, respectively. The raw data and calculations can be found in Table S1.

A possible explanation could be that sunscreen B and C contained UVA-filter. UVA filters are only responsible for 0.5% (UVB is 99.5% responsible) of the erythema action spectra, and therefore not fully measured by the Mansur method. In order for a sunscreen to be denoted as a broadband protector, the UVA-PF/SPF ratio must be at least 1/3 of the labelled SPF, which means that at least 1/3 of the claimed SPF of sunscreen B and C is not being measured by the Mansur method. This was confirmed by the test-report on the commercial products (sunscreen B and C), which presented details on the SPF, UVA-PF, and ratio of UVA-PF/SPF of the sunscreens (data not shown). From the test-report, sunscreen B had a SPF of 27, which is essentially equal to the combined SPF (Mansur) + UVA-PF. Sunscreen C had a reported SPF of 43.3 when combining SPF (Mansur) and UVA-PF. The test-report revealed that the UVA-PF of sunscreen B and C were 11 and 22, respectively. When these numbers were added to the SPF from the Mansur method (Table 4), the total SPF were in fact in very similar to the claimed SPF. Thus, SPF measured by Mansur does not include protection against UVA rays [25].

A similar SPF evaluation was performed by Ácsová et al. [11]. This study tested six commercial sun oils with a SPF of 6 to 30. Their results were in close agreement with the claimed SPF for all six oils. All oils contained UVB-filters, but only two oils also contained UVA-filter (bis-ethylhexyloxyphenol) in low amounts (1–5%). However, it was not mentioned whether these two sun oils were marked as broadband absorbers.

The benefits of the Mansur method are that it utilizes low-cost solvents, is fast, and only requires conventional laboratory spectrophotometers. The Mansur method can therefore be useful as a rough prediction during product development, particularly for products containing only UVB-filters. If UVA-filters are present, the application of the COLIPA UVA-PF method is necessary. For accurate SPF-determination, the photo-protective properties must be confirmed in an accredited laboratory through the ISO 24444:2019 in vivo method [19]. Previously, the Mansur method have been applied to determine the SPF of wheat germ oil (SPF 22.40), carrot seed oil (SPF 18.80) and olive oil (SPF 9.3) in a study by Suryawanshi [30]. Moreover, Kasitowati et al. [31] applied the Mansur method to determine the SPF of methanol extract (SPF 19.65) and ethyl acetate (SPF 26.45) from red algae *Eucheuma* sp. 

Reviewing some studies, indicates simplification of the Mansur method and equation and incorrect use of in vitro SPF screening. This simplification is mainly due to wrong dilutions during sample preparation, and thereby an overestimation of the SPF. Kaur & Saraf [32] determined SPF of different oils by the Mansur method, using a dilution factor of 0.1% instead of 0.02%, whereby the SPF was overestimated five-fold. In another study by Suryawanshi [30], the concentration of oil was not listed, but according to the extremely high absorbance values measured (Abs > 3) for wheat germ oil and carrot oil in particular, it is likely that the oil was not diluted correctly. Kasitowati et al. [31] applied the Mansur method to determine the SPF of extract from *Euchea* sp. (Rhodophyceae) and reported SPF of 19.65 and 26.45 for methanol and ethyl acetate extracts, respectively. They did not mention whether or how they diluted the extracts. Hence, assuming that they analyzed the extract without diluting it instead of diluting to the reference concentration of 0.02%, this could potentially mean a 5000 fold overestimation of the determined SPF value. Hence, it is important to be aware of this since the Mansur method is not a standardized method yet to determine SPF.

#### 2.3.3. Sun Protection Factor Seaweed Enriched Lotions

Based on our tests with commercial sunscreens, the Mansur method was applied to determine SPF in seaweed enriched lotion (SWE-L). SWE-Ls were prepared by adding two different concentrations of freeze-dried FV-WE (10 and 20%) and FV-EE (14.1 and 28.2%) to a cream base. Typically, commercial UV-filters are also added in this range (4–25%) [33]. Both FV-WE and FV-EE-enriched lotions were visibly colored. Without the extracts added the lotion was white, addition of FV-WE gave a distinct brown color, whilst FV-EE gave a brown-green tint of the lotion as shown in Table 5 (color data not shown).

All four SWE-L presented negligible photo-protective capacity (Table 5). A linear correlation between extract concentration and SPF (dose/response) was observed. Although adding higher concentrations would have been possible, this would likely never have been commercially acceptable. It is also highly unlikely that the SPF would reach any significant values through the addition of seaweed extract produced in this study.

Ácsová et al. [11] also found in vitro SPF < 1 for numerous vegetable oils, whilst in vivo results were all SPF > 2. The difference between in vivo and in vitro results underlines the importance of this method only being utilized as screening, since an “ingredient” can protect against erythema by other means than just absorbing UV-rays. Ácsová et al. [11] explained the in vitro/in vivo discrepancy as being due to the presence of accompanying substances in the oil (e.g., tocopherols, carotenoids, phytosterols) that exert anti-inflammatory, antioxidant and anti-erythema properties, essentially protecting towards reddening of the skin in other ways than solely by absorbing UV-rays.

Although this suggests that the in vivo results could be slightly higher, the gap between the found and desired results are substantial. After much consideration, the most likely explanation to the low SPF is the fact that the inorganic and organic filters used in commercial sunscreens are pure chemicals, i.e., each molecule is specifically designed or chosen to absorb/reflect light at a specific wavelength. In contrast, in seaweed extracts, a ballpark value of 1 out of 10,000 chemicals absorb UV-light, whilst the remainder are other chemicals/compounds/solvent. Therefore, the concentration of UV-absorbing compounds is simply too low to have any significant effect. 

However, the absorption spectra displayed some absorption in the UVA-range, indicating the presence of UVA-absorbing compounds. As the UVA-PF is not measured through the Mansur-method, the photo protective capacity is expected to be somewhat higher than what is presented in Table 5. Additionally, if the theory presented by Ácsová et al. [11] regarding the in vitro/in vivo discrepancy is correct, it is also possible that the in vivo results could be somewhat higher, due to other properties than simply UV-absorbance.

### 2.4. Determining Effective Solar Absorbed Radiation—Photo Protecting Index

FV-WE (G1 and G4) showed higher SPF by the Mansur method and also some absorption outside the UVB range. To get more information about the UV protecting properties of FV-WE (G1 and G4) the effective solar absorbed radiation (%ESAR) and the extract photo protection index (EPI) were measured and calculated. Table 6 shows %ESAR of FV-WE (G1 and G4).

The %ESAR was proposed in this study as an indicator of extract photoprotection properties, considering the retained radiation (avoidance of the occurrence of biological response) from 0 to 100%. %ESAR shows the action of the seaweed extracts on UV wavelengths that induce different biological responses, in this case erythema, PPD, elastosis and photo aging. Whereas erythema is caused 99.5% by UVB and only 0.5% by UVA (0.3% UVAI and 0.2% UVAII), UVB is to a lower degree responsible for elastosis (63.4%), photo aging (3.6%) and PPD (2.9%), which are mainly caused by UVA rays. 

%ESAR of FV-WE G1 and G4 increased with concentration indicating a concentration dependent response, and ranged between 20.9 to 37.7% for erythema, 8.1 to 13.2% for PPD, 7.3 to 11.8% for elastosis, and 8.2 to 13.5% for photo aging. Thus, higher %ESAR values indicate greater potential of the extracts to absorb certain wavelengths, preventing and/or reducing the induction of the biological response associated with the absorbed wavelength.

The extracts in the present study in general showed higher %ESAR than *Carpodesmia tamariscifolia* (5.6%), another brown seaweed, and similar %ESAR against erythema as for *Porphyra umbilicalis* (red), *Sargassum vulgare* (brown), and *Ulva lactuca* (green) at 2 mg cm^−1^ dw [25]. However, for other responses like PPD, elastosis and photo-aging FV-WE had approximately three times lower %ESAR compared to *Porphyra umbilicalis* and *Sargassum vulgare*. This indicates that FV-WE are lacking some of the important UVA protecting compounds for these biological responses, like MAAs. 

EPI can be related to SPF (erythema) and UVA-PF (PPD), with a maximum value of 50. In Table 7 EPI of FV-WE G1 and G4 are shown. EPI for erythema was slightly higher than for PPD at the same concentrations, ranging between 1.2 and 1.6 and 1.2 and 1.3, respectively, and no clear concentration dependency was observed. Schneider et al. [25] found hyperbolic trend responses with application of different action spectra, showing EPI values of up to 50. However, this was not possible to obtain in the present study.

FV-WE G1 and G4 were also added to o/w skin care emulsion (10% *w*/*w*), similar to the seaweed enriched lotions (SWE-L) used in the Mansur method. In Table 8 %ESAR and EPI of SWE-L with FV-WE G1 and G4 added are shown. Values for %ESAR and EPI were similar to those found for the crude extract when evaluated in the highest concentration (5 mg cm^−1^).

The results of the SPF evaluated by EPI is not in correlation with the results obtained from the Mansur method. Whereas the Mansur method obtained a SPF of approximately 0.5 when the FV-WE was added in concentration of 10%, EPI showed SPF of approximately 1.4. Hence, there are some uncertainties related to EPI, which is probably higher when the %ESAR is low, which was also why the concentration dependency of EPI of the crude extracts was not clear (Table 7). However, %ESAR provides information on the photo protection properties of FV-WE for different biological responses, which the Mansur method is lacking. Therefore, this method is highly relevant as a screening tool.

Soleimani et al [34] studied the phlorotanins from brown alga *Polycladia myrica* as cosmeceuticals using similar method for determining EPI in the present study. They successfully increased the concentration of phlorotannins in ethanolic extracts (50:50 water:ethanol) by ethyl acetate precipitation, from 2.7  ±  0.1 to 12.1  ±  0.1 phloroglucinol equivalents per gram of dry weight (DW). The ethyl acetate fraction obtained high degree of photoprotection when added in cream formulations, SPF of 31.79  ±  4.73 and UVA-PF of 24.67  ±  4.03, respectively. This indicated confirms that further concentrating phlorotannins is needed to improve the photoprotecting properties of these types of extracts from *F. vesiculosus*.

## 3. Materials and Methods

### 3.1. Seaweed Material

Brown algae *Fucus vesiculosus* was harvested by hand in August/September 2019 at Begtrup vig (56°9′54.6″ N 10°28′24.7″ E) east of Aarhus, Denmark, by Organic Seaweed A/S (OS). Four different growth depths were chosen (Table 1). After harvest, the seaweed was rinsed with water to remove epiphytes, before air dried (drying facilities at OS). The seaweed was stored in sealed plastic bags at room temperatures (dry and dark) until extraction (storage stability of 2 years).

### 3.2. Extraction of Phlorotannins

The aim was to obtain heat and storage stable (−20 °C for 12 months) extracts from *F. vesiculosus* with a high content of phlorotannin using solid-liquid extracts (SLE). The extraction procedure was in accordance with Wang et al. [26] with modifications. The extraction solvents used were water and ethanol (67% *v*/*v*). Ethanol for efficient extraction of phlorotannins was used in accordance with Hermund et al. [9]. The air-dried seaweed was grinded to a powder (<1 mm). A quantity of 5 g of powdered seaweeds were added to 100 mL water or ethanol (67% *v*/*v*), and vigorously shaken for 30 s. The extraction was performed over 24 h at 20 °C in the dark by using a platform shaker (Heidolph Instruments, Unimax 2010, Schwabach, Germany) at 125 rpm. Subsequently, the extracts were centrifuged for 10 min at 18 °C and 1665× *g*. The extract was decanted, and the supernatant collected. The residue was re-extracted (same conditions as above), and the supernatants were pooled. Two types of extracts were obtained; a water extract (FV-WE) and an ethanolic extract (FV-EE) of each group from giving a total of 8 extracts.

The procedure was performed for all four samples of seaweed, with both solvents. To assess the reproducibility of the method, the procedure was performed in duplicates. The reproducibility of SLE was evaluated by determining total phenolic content for the duplicates, and no significant difference (*p* < 0.05) was found between the extracts of the same group. The extracts were pooled and lipophilised or stored at −80 °C until analysis. The dry matter of the liquid extracts was from 0.9–1.1% in FV-WE and from 0.7–0.8% in FV-EE. Dry matter is used when calculating the results per dry weight (dw).

### 3.3. Characterization of Extracts

#### 3.3.1. Phlorotannin Content

The phlorotannin content was estimated by determining the total phenolic content (TPC) of the extracts using a modified version of the method performed by Farvin & Jacobsen [7]. The liquid extracts were diluted 10 times before analysis. To 100 μL of diluted sample 0.75 mL Folin Ciolcalteu phenol reagent (10% *v*/*v* in water) was added and mixed. After 5 min, 0.75 mL sodium-carbonate solution (7.5% Na_2_CO_3_ *w*/*v* in water) was added and mixed. The reaction was incubated for 90 minutes in room temperature in the dark. The absorbance was measured at 725 nm by a UV-vis spectrophotometer (Shimadzu UV mini−1240, Duisburg, Germany). Gallic-acid (GA, 2,3,4-trihydrobenzoic acid) was used for quantification (calibration curve: 0–250 μg mL^−1^). The results are expressed as grams of gallic acid equivalents (GAE) in 100 g dw (g GAE 100 g^−1^ dw, *n* = 3).

#### 3.3.2. Antioxidant Properties

DPPH radical scavenging capacity. The radical scavenging capacity of the extracts was quantified using 2,2-diphenyl-1-picrylhydrazyl (DPPH), by applying the method described by Yang et al. [35] modified for use in a 96-well microplate. 100 μL extract solution (8 different dilutions of the extract) and 100 μL 0.1 mM DPPH solution (in 96% ethanol) were mixed in the microtiter plate. Reaction mixtures were incubated for 30 min at room temperature in the dark. The absorbance was measured at 517 nm using a microplate reader (BioTek Eon, BioTek Instruments Inc., Winooski, VT, USA) and Gen5 2.09 data analysis software. BHT was included as a positive control (63% inhibition in concentration of 0.2 mg mL^−1^). The results are expressed as EC50 μg dw mL^−1^ (dw in extract mL^−1^ liquid extract) (*n* = 3).

Ferrous iron chelating ability. The iron chelating ability was determined using the method described by Farvin et al., [36]. One hundred μL extract solution (8 different dilutions of the extract) were added to the microtiter plate together with 110 μL distilled water. Twenty μL 0.5 mM ferrous chloride tetrahydrate were added, and mixed. The reaction mixture was incubated for 3 min before 20 μL 2.5 mM ferrozine were added. The reaction mixture was incubated for 10 min at room temperature in the dark before the absorbance was measured at 562 nm. EDTA was included as a positive control (53% inhibition in concentration of 0.06 mM). The results are expressed as EC50 μg dw mL^−1^ (dw in extract mL^−1^ liquid extract) (*n* = 3).

### 3.4. Screening of UV Protective Capacity of Natural Extracts and Sunscreen Formulations

#### 3.4.1. Absorption Spectrum

Two groups, G1 and G4 were selected for further analysis based on the screening. The absorption spectrum of FV-WEG1, FV-WEG4, FV-EEG1 and FV-EEG4 was obtained using a quartz cuvette and measured spectrophotometrically (Shimadzu UV mini-1240, Duisburg, Germany). The spectrum was made in duplicates for each group and solvent type. Two spectral windows were made: 280–400 nm (UVB + UVA) and 400–700 nm (visible range) with a resolution of 1 nm.

#### 3.4.2. Mansur Method for Determining Sun Protection Factor (SPF)

##### Mansur Method on Commercial Sunscreens

The Mansur method was tested using three commercial sunscreens labelled SPF 15, 25 and 45 (Table 3), with the two latter providing broadband protection. 

For this method, 1 g of freshly opened sunscreen was weighed and transferred to a 100 mL volumetric flask and mixed with 50 mL 96% ethanol using a magnetic stirrer until fully dissolved, and then degassed in ultrasonic bath for 5 min. Fifty mL 96% ethanol were added and mixed to create 1% stock solution (SS). The SS was passed through a Whatman filter paper 1, rejecting the first 10 mL. A 1 mL aliquot was added to a 50 mL volumetric flask and diluted to volume with 96% ethanol and mixed well, creating the solution at reference concentration (SRC) of 0.20 mg/mL (1:50 dilution of the 1% SS). The absorption spectrum of the SRC was obtained in the range of 250–400 nm with increments of 1 nm (Shimadzu UV mini-1240, Duisburg, Germany), using a quartz cuvette and ethanol as blank. The absorption data were obtained in 5 nm increments between 290 and 320 nm. The results were calculated using the Mansur equation (Equation (1)) (*n* = 3).

##### Mansur Method on Seaweed Enriched Lotions

Oil-in-water (o/w) lotion was provided by Melissa Organic Skincare to produce seaweed-enriched lotion (SWE-L). Lyophilized FV-WE of G1 and G4 were added to the cream base in 20% concentrations. To account for the lower dry matter in the FV-EE (FV-EE ≈ 0.7% DM, FV-WE ≈ 1% DM), lyophilized FV-EE of G1 and G4 were added to the cream base in concentrations of 28.2%. The cream base with and without seaweed extracts were analyzed using the Mansur method as described above. To evaluate the relationship between concentrations of seaweed extract and SPF, different dilutions were made on the SRC of the four different SWE-L.

#### 3.4.3. Effective Solar Absorption Radiation (%ESAR) Ratio and Extract Photoprotection Index (EPI) 

The effective solar absorption radiation (%ESAR) and extract photoprotection index (EPI) of seaweed extracts were determined based on the methodology from Schneider et al. [25]. The %ESAR and EPI determination were only performed for FV-WE group G1 and G4, which were selected due to the results of the screening by Mansur method, showing an increased SPF when these two FV-WE were added to lotions. 

EPI and %ESAR were calculated by applying 800 μL of different dilutions of the concentrated FV-WE (start concentration 10 mg dry extract in 1 mL dH_2_O). On the rough side of polymethylmethacrylate (PMMA) plates (roughness Ra, 4.5–5.2 μm; dimension: 5 cm × 5 cm × 0.25 cm, 25 cm^2^; Schönberg GmbH & Co. KG, Cochem, Germany), which yielded final concentrations per plate area of 1.25, 2.5 and 5 mg of dry FV-WE per cm^2^ (mg dw cm^−2^). 

Moreover, FV-WE G1 and G4 extracts were incorporated in o/w lotions at a concentration of 10% (*w*/*w*) and ESAR (%) and different photoprotection factors were calculated (SPF, UVAPF and BEPFs). A total of 30 mg of the lotions were applied on the plates. From here on, the procedure was as described by Schneider et al. [25]. The transmittance through the plates were determined using a spectrophotometer (UV-2700i, Shimadzu, Duisburg, Germany) with an integrated sphere. Three plates (*n* = 3) were measured for each sample. The parameter %ESAR, EPI and BEPFs were calculated by applying action spectra of different biological responses driven by UV (erythema, PPD, elastosis and photo aging) as described by Schneider et al. [25]. 

### 3.5. Data Treatment

For comparison of TPC and antioxidant activity between groups (growth depth) and solvent type (water and 67% ethanol), two-way analysis of variance (ANOVA) was performed with Tukey’s multiple comparison test. The results are designated as significant when *p* < 0.05. The software used for statistical analysis was Graphpad prism 9 (Graphpad Software Inc., San Diego, CA, USA).

The main variance in the data set was detected using Principal Component Analysis (PCA), using the Unscrambler 11.0 software (Camo, Oslo, Norway). A PCA allows for detection of similarities and differences between the different samples (groups) in a score plot, whereas correlation between the measured variables (TPC and antioxidant assays) is visualized in a correlation loadings plot. All data were mean centered and scaled to equal unit variance prior to PCA.

## 4. Conclusions

It was hypothesized that the growth depth would affect the phlorotannin content, antioxidant capacity and especially the photo protective properties of *F. vesiculosus*, and that the phlorotannin content and antioxidant capacity would increase with increasing level of solar exposure (more exposure at lower growth depth). However, growth depth did not significantly influence the TPC or antioxidant activity, nor the photo-protective capacity of the two types of *F. vesiculosus* extracts obtained (FV-WE and FV-EE). The most likely explanations to this are the low variation in growth depth, as well as the multitude of biotic and abiotic factors influencing the biochemical properties of seaweed.

It was confirmed that the applied extraction method (SLE) using water or 67% ethanol successfully extracted phenolic compounds. The type of solvent did not significantly impact the TPC of the extracts, but ethanol was more efficient in extracting components exerting DPPH radical scavenging capacity, whilst the FIC was higher in the FV-WE. The TPC correlated with DPPH and not FIC, indicating that the FIC is influenced by other co-extracted components, and not only phenolic compounds. It can be concluded that *F. vesiculosus* is a rich source of natural antioxidants and a source of topical antioxidants for cosmeceutical purposes.

Finally, it was hypothesized that these phlorotannin-rich extracts provide photo-protective activity and can be utilized as an ingredient to increase the SPF of sunscreens. The results showed that the photo-protective capacities presented by FV-WE and FV-EE in cream base are negligible by the Mansur method. On the other hand, FV-WE had photo protecting properties against some (mainly) UVA induced biological responses. However, EPI still showed poor photo protecting properties. The most likely explanation is that the photo-protective components are present in too low concentration, and that the distance between them in the cream will therefore be too large. It can therefore be concluded that employing SLE of *F. vesiculosus* is insufficient in yielding an extract with any substantial SPF, without additional fractionation or purification.

Lastly, this paper provides correct description of how to use the Mansur method, which in past research have been misinterpreted resulting in overestimation of SPF. Hence, this method can be used as in vitro screening tool, e.g., for new seaweed-based cosmeceutical ingredients, providing information on the UVB protection properties.

## Figures and Tables

**Table 1:** Overview of the four seaweed groups, their growth depth, harvesting time, quality assessment and observed color.

Group	Growth Depth	Harvesting Time	Quality Assessment	Color
G1	15–30 cm	20 August 2019	Low	Yellow/brown
G2	20–30 cm	7 September 2019	High	Yellow/brown
G3	30–50 cm	7 September 2019	High	Green
G4	50–70 cm	7 September 2019	High	Yellow/brown

**Table 2:** Total phenolic content (g GAE 100 g^−1^ dw), DPPH radical scavenging capacity (EC50 (µg mL^−1^)) and Ferrous iron chelating ability (EC50 (µg dw mL^−1^)) of FV-WE and FV-EE from different water depth (G1, G2, G3 and G4). Mean ± SD (*n* = 3).

Water Depth	TPC		DPPH		FIC	
	g GAE 100 g^−1^ dw		EC_50_ (µg dw mL^−1^)		EC_50_ (µg dw mL^−1^)	
Group	FV-WE	FV-EE	FV-WE	FV-EE	FV-WE	FV-EE
G1 (15–30 cm)	10.4 ± 0.8 ^ax^	11.7 ± 0.3 ^ax^	33.3 ± 1.1 ^ax^	16.3 ± 0.5 ^ay^	360.0 ± 66.3 ^ax^	824.6 ± 70.5 ^ay^
G2 (20–30 cm)	13.7 ± 1.7 ^bx^	14.0 ± 0.6 ^bx^	31.1 ± 3.6 ^ax^	15.5 ± 2.4 ^ay^	365.1 ± 31.3 ^ax^	1052.4 ± 43.5 ^ay^
G3 (30–50 cm)	6.9 ± 1.1 ^cx^	7.8 ± 0.4 ^cx^	76.3 ± 2.5 ^bx^	30.9 ± 0.8 ^by^	340.4 ± 24.7 ^ax^	754.2 ± 29.5 ^ay^
G4 (50–70 cm)	11.9 ± 0.6 ^ax^	11.2 ± 0.9 ^ax^	35.5 ± 3.2 ^ax^	20.1 ± 1.3 ^ay^	311.2 ± 71.7 ^ax^	635.3 ± 38.0 ^ay^

x,y indicates significance (*p* < 0.05) between FV-WE and FV-EE within the same group (G1–G4) and analysis (vertical). a–c indicates significance (*p* < 0.05) between groups (G1–G4) within the same analysis (horizontal).

**Table 3:** Characteristics of three commercial sunscreens (A, B, C) used for testing the Mansur method including claimed SPF, type of UV filter.

Sample	Claimed SPF	UV Filters Concentration, Sun filter (INCI Name, Filter Type and O = Organic or IO = Inorganic)	Broadband Protection
A	15	Non given	No
B	25	5% Diethylamino hydroxybenyl (UVA filter) (O) 9% Ethylhexyl triazone (UVB filter) (O)	Yes
C	45	10% Diethylamino hydroxybenyl (UVA filter) (O) 3% Ethylhexyl triazone (UVB filter) (O) 3% Diethylhexyl butamido triazone (O)	Yes

**Table 4:** Sun protection factor of claimed vs. in vitro SPF results obtained using the Mansur method of three commercial sunscreens (A, B, C). Mean ± SD (*n* = 3).

Sample	Claimed SPF	Measured SPF by Mansur Method	Measured vs. Claimed SPF
A	15	16.3 ± 0.1	108%
B	25	16.0 ± 0.0	64%
C	45	21.3 ± 0.0	47%

**Table 5:** Sun protection factor (SPF) of seaweed enriched lotions with FV-WE or FV-EE (G1 and G4) added in different concentrations (0–20% *w*/*w* or 0–28% *w*/*w*, respectively). Mean ± SD (*n* = 3).

Extract	Group	Extract Concentration (% *w*/*w*)	SPF	Color of the Lotion when 10% FV-WE or 14% FV-EE Was Added
Control	-	0	0.2 ± 0.0	
FV-WE	G1	10	0.5 ± 0.0	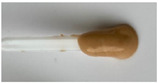
		20	1.1 ± 0.0	
	G4	10	0.6 ± 0.1	
		20	1.1 ± 0.0	
FV-EE	G1	14	0.2 ± 0.1	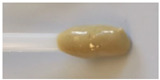
		28	0.5 ± 0.0	
	G4	14	0.2 ± 0.0	
		28	0.5 ± 0.1	

**Table 6:** Effective solar absorption radiation (ESAR, in %) by FV-WE G1 and G4, in relation to erythema, PPD, elastosis and photo aging. Different extract concentrations (1.25, 2.5 and 5 mg cm^−1^) were tested on the plates. Mean ± SD (*n* = 8). PPD = Persistent pigment darkening.

	Concentration mg DE Plate^−1^	Erythema	PPD	Elastosis	Photo Aging
**FV-WE G1**	5	28.6 ± 2.8	10.3 ± 1.4	9.2 ± 1.3	10.5 ± 1.4
	2.5	26.1 ± 2.2	10.1 ± 1.2	9.1 ± 1.1	10.2 ± 1.2
	1.25	21.7 ± 3.1	9.0 ± 1.4	8.1 ± 1.3	9.0 ± 1.4
**FV-WE G4**	5	36.7 ± 1.1	13.2 ± 0.6	11.8 ± 0.6	13.5 ± 0.6
	2.5	27.4 ± 4.4	10.5 ± 1.8	9.5 ± 1.6	10.7 ± 1.8
	1.25	20.9 ± 1.5	8.1 ± 0.6	7.3 ± 0.6	8.2 ± 0.6

**Table 7:** Extract photo protection index (EPI) by FV-WE G1 and G4, related to erythema, PPD, elastosis and photo aging. Different extract concentrations (1.25, 2.5 and 5 mg/cm) were tested on the plates. Mean ± sd (*n* = 8). PPD = Persistent pigment darkening.

	Concentration mg DE Plate^−1^	Erythema (SPF)	PPD (UVA-PF)	Elastosis	Photo Aging
**FV-WE G1**	5	1.4 ± 0.1	1.3 ± 0.0	1.3 ± 0.0	1.3 ± 0.0
	2.5	1.4 ± 0.0	1.2 ± 0.0	1.3 ± 0.0	1.2 ± 0.0
	1.25	1.3 ± 0.1	1.2 ± 0.0	1.2 ± 0.0	1.2 ± 0.0
**FV-WE G4**	5	1.6 ± 0.0	1.3 ± 0.0	1.4 ± 0.0	1.4 ± 0.0
	2.5	1.4 ± 0.1	1.3 ± 0.1	1.3 ± 0.1	1.3 ± 0.1
	1.25	1.3 ± 0.0	1.2 ± 0.0	1.2 ± 0.0	1.2 ± 0.0

**Table 8:** Effective solar absorption radiation (ESAR, in %) and Extract photo protection index (EPI) of different photoprotection factors (SPF, UVAPF and BEPFs against elastosis and photo aging) of seaweed enriched lotions with 10% FV-WE G1 or G4 added. Mean ± SD (*n* = 8). PPD = Persistent pigment darkening, SPF = sun protection factor, pf = protection factor.

	Seaweed Enriched Lotion	Erythema (SPF)	PPD (UVA-PF)	Elastosis	Photo Aging
**%ESAR**	10%-FV-WE G1	22.3 ± 4.4	5.5 ± 1.5	4.8 ± 1.3	5.8 ± 1.6
	10%-FV-WE G4	34.9 ± 5.2	9.8 ± 1.8	8.6 ± 1.6	10.4 ± 1.9
**EPI**	10%-FV-WE G1	1.3 ± 0.1	1.1 ± 0.0	1.1 ± 0.0	1.1 ± 0.0
	10%-FV-WE G4	1.5 ± 0.1	1.2 ± 0.1	1.3 ± 0.1	1.3 ± 0.1

## Data Availability

The datasets generated during and/or analyzed during the current study are available from the corresponding author on reasonable request.

## Supplementary Materials

The following supporting information can be downloaded at https://www.mdpi.com/article/10.3390/md20110687/s1, Figure S1: Absorption spectra in the UV-region (280–400 nm), Figure S2: Absorption spectra in the visible light region (400–700 nm), Figure S3: Heat stability of FV-WE, Figure S4: Storage stability of FV-EE and FV-WE, Table S1: Raw data and calculation of SPF of commercial sunscreens.
