# Supplementary material for: Screening for New Cosmeceuticals from Brown Algae Fucus vesiculosus with Antioxidant and Photo-Protecting Properties
Source: Mar Drugs. 2022 Oct 31;20(11):687. doi: 10.3390/md20110687 (PMC9697279; doi:10.3390/md20110687)
Supplement: Supplementary file 1 [file marinedrugs-20-00687-s001.zip › marinedrugs-1935212-supplementary.pdf]

Supplementary material

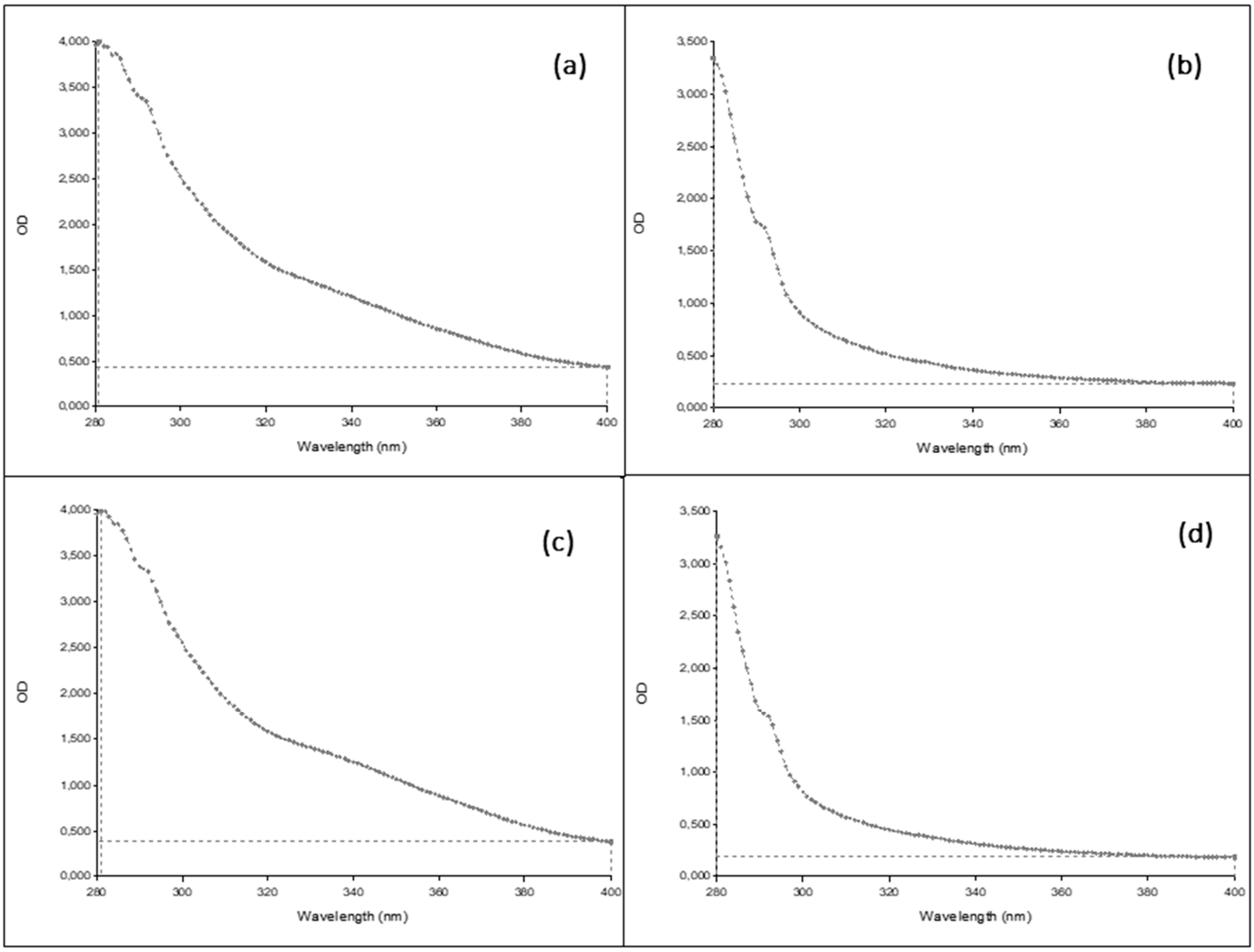

Figure S1. Absorption spectra in the UV-region (280-400 nm) of FV-WEG1 (a), FV-WEG4 (c), FV-EEG1 (b) and FV-EEG4 (d). The extracts are diluted 1:2 in water or 67 % ethanol.

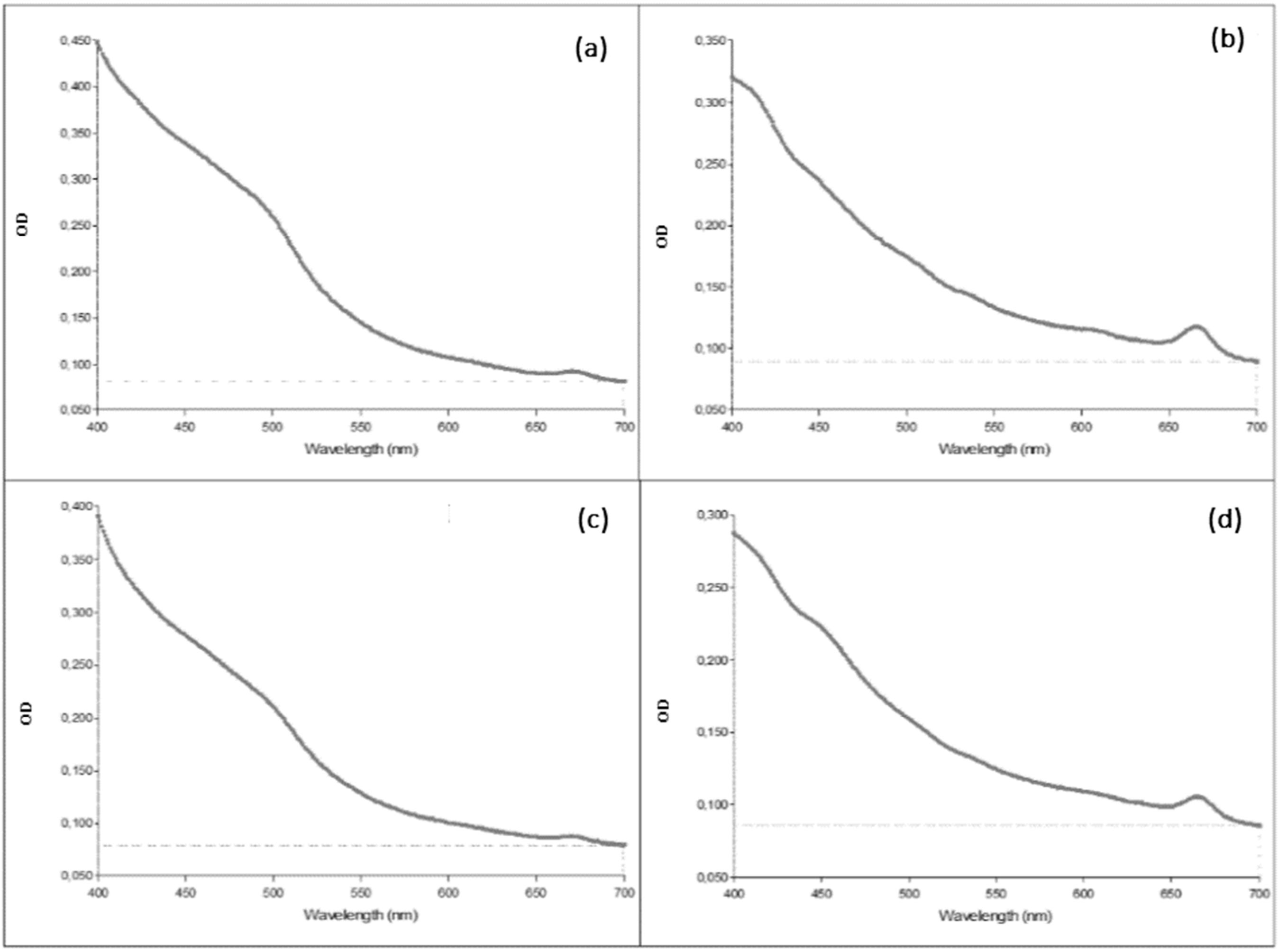

Figure S2. Absorption spectra in the visible light region (400-700 nm) of FV-WEG1 (a), FV-WEG4 (c), FV-EEG1 (b) and FV-EEG4 (d). The extracts are diluted 1:2 in water or 67% ethanol.

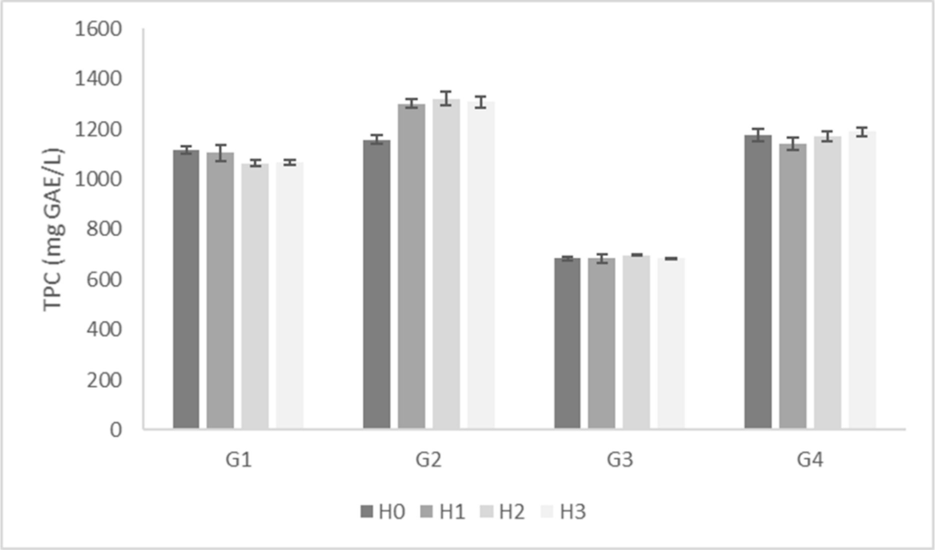

Figure S3 Heat stability of FV-WE

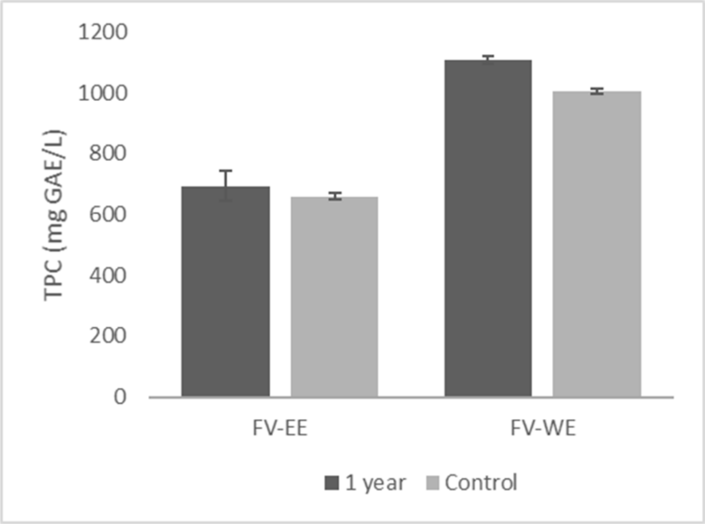

Figure S4. Storage stability of FV-EE and FV-WE.

Table S1. *Raw data and calculation of SPF of commercial sunscreens (A (SPF15), B (SPF25) and C (SPF45)) using the Mansur method. (B and C have a dilution factor of 10)*

			Rep 1			Rep 2			Rep 3		
**Pro-duct**	**Wave-lenght nm**	**EE*I**	**Abs**	**Abs*EE*I**	**SPF**	**Abs**	**Abs*EE*I**	**SPF**	**Abs**	**Abs*EE*I**	**SPF**
A	290	0,015	0,91	0,01365		0,896	0,01344		0,9	0,0135	
	295	0,0817	1,174	0,09591		1,159	0,0946903		1,164	0,0950988	
	300	0,2874	1,491	0,42851		1,476	0,4242024		1,48	0,425352	
	305	0,3278	1,71	0,56053		1,698	0,5566044		1,702	0,5579156	
	310	0,1864	1,875	0,3495		1,861	0,3468904		1,863	0,3472632	
	315	0,0839	1,944	0,16310		1,941	0,1628499		1,944	0,1631016	
	320	0,018	1,483	0,02669		1,499	0,026982		1,499	0,026982	
				**1,6379128**	**16.38**		**1,6256594**	**16.26**		**1,6292132**	**16.29**
B	290	0,015	0,893	0,013395		0,893	0,013395		0,894	0,01341	
	295	0,0817	1,146	0,0936282		1,145	0,0935465		1,147	0,0937099	
	300	0,2874	1,452	0,4173048		1,453	0,4175922		1,455	0,418167	
	305	0,3278	1,668	0,5467704		1,67	0,547426		1,673	0,5484094	
	310	0,1864	1,827	0,3405528		1,829	0,3409256		1,835	0,342044	
	315	0,0839	1,906	0,1599134		1,912	0,1604168		1,913	0,1605007	
	320	0,018	1,488	0,026784		1,495	0,02691		1,497	0,026946	
				**1,5983486**	**15.98**		**1,6002121**	**16.00**		**1,603187**	**16.03**
C	290	0,015	1,278	0,01917		1,278	0,01917		1,276	0,01914	
	295	0,0817	1,605	0,1311285		1,603	0,1309651		1,601	0,1308017	
	300	0,2874	1,968	0,5656032		1,965	0,564741		1,964	0,5644536	
	305	0,3278	2,199	0,7208322		2,2	0,72116		2,2	0,72116	
	310	0,1864	2,412	0,4495968		2,411	0,4494104		2,409	0,4490376	
	315	0,0839	2,449	0,2054711		2,446	0,2052194		2,45	0,205555	
	320	0,018	1,903	0,034254		1,898	0,034164		1,908	0,034344	
				**2,1260558**	**21.26**		**2,1248299**	**21.25**		**2,1244919**	**21.24**
